# Radical Scavenging Actions and Immunomodulatory Activity of *Aronia melanocarpa* Propylene Glycol Extracts

**DOI:** 10.3390/plants10112458

**Published:** 2021-11-15

**Authors:** Kseniya Bushmeleva, Alexandra Vyshtakalyuk, Dmitriy Terenzhev, Timur Belov, Andrey Parfenov, Natalia Sharonova, Evgeniy Nikitin, Vladimir Zobov

**Affiliations:** 1 Laboratory for Plant Raw Material Conversion for Organic Farming, Federal State Budgetary Institution of Science Federal Research Center, Kazan Scientific Center of Russian Academy of Sciences, 2/31 Lobachevskogo Str., 420111 Tatarstan, Russia; dmitriy.terenzhev@mail.ru (D.T.); belofftimur@mail.ru (T.B.); lapanovich@mail.ru (N.S.); berkutru@mail.ru (E.N.); 2 A.E. Arbuzov Institute of Organic and Physical Chemistry, Kazan Scientific Center, Russian Academy of Sciences, Arbuzov Str. 8, 420088 Kazan, Russia; alex.vysh@mail.ru (A.V.); aimt66@gmail.com (A.P.); vz30608@mail.ru (V.Z.)

**Keywords:** *Aronia melanocarpa*, black chokeberry, bioactive compounds, extracts, method of preparing raw materials, antioxidant capacity, immunomodulatory activity

## Abstract

Researchers are attracted to the wide-ranging, useful components in *Aronia melanocarpa* berries. They are searching for the most effective ways to extract the active substances that can enhance the body’s protective properties. The current study presents detailed information about the extracts from *A. melanocarpa* fruits frozen and dried under mild conditions and their chemical composition. In Wistar rats with induced immunosuppression, the effect of chokeberry fruit extracts on the leukocyte formula, phagocytic activity, and cytokine system was studied. It was shown that the *A. melanocarpa* frozen fruit extract contains more anthocyanins, sugars, and ascorbic acid, and has a more pronounced antioxidant activity determined by the ability to bind APPH-radicals. Moreover, the extract showed membrane-protective and cytoprotective properties against RPMI-1788 cell line. The extract from dried raw material shows a higher antioxidant activity due to the ability to bind DPPH-radicals. It was revealed that extracts from *A. melanocarpa* fruits promote rapid immune system recovery in rats, normalize the leukocyte count, and improve monocyte and neutrophil phagocytic indicators. Research on the cytokine profile revealed that the anti-inflammatory properties in *A. melanocarpa* extracts were more pronounced in dried extracts. For several cytokines, a normalization of quantity was noted.

## 1. Introduction

The increased usage of numerous chemicals in everyday products, as well the consequent human exposure to various health hazards, has sparked growing interest in possible ways to protect against their adverse effects and associated disease. Compounds that interact with the immune system to enhance or suppress specific immune responses can be classified as immunomodulators or biological response modifiers [1]. Immunomodulatory activity has been identified in a wide variety of plant extracts used in folk medicine, which provides a rational explanation for their medicinal use [2].

Some components in the plants’ raw materials, such as galactolipids, proanthocyanidins, and flavonoids, can influence the plants’ immunomodulatory properties [3,4]. Cyanidin-3-O-glucoside, which is found in plant extracts, has anti-inflammatory, neuroprotective, antithrombotic, and epigenetic properties. Quercetin and rutin have significant cytoprotective potential and cause high Keap1-Nrf2 activity. The significant cytoprotective ability of flavonoids can be explained by the numerous antioxidant protein expressions induced by Keap1-Nrf2 pathway activation [5]. Quercetin and the more readily accessible rutin have been shown to protect against diabetes, cardiovascular disease, inflammation, cancer, as well as nerve damage and retinal cells. Anthocyanins and anthocyanidins have immunomodulatory and cardio-protective properties. Flavonic acids exhibit anti-inflammatory, antioxidant, hypotensive, antihyperglycemic, and cellular protective actions [6]. Lipid components, polyphenolic acids, and flavonones provide mucosal cytoprotection by increasing the prostaglandin production as well as glycogen and glycoproteins such as mucin. These compounds cause vascular endothelial growth factor (VEGF) activation, angiogenesis enhancement, blood flow enhancement, hexosamine, the increase in sialic acid, collagen fiber proliferation, and epidermal growth factor receptor activation. In general, they are involved in the prevention and treatment of gastric ulcers [7].

*Aronia melanocarpa* fruit is valued as an excellent source of antioxidants, especially polyphenols such as phenolic acids (neochlorogenic and chlorogenic acids), and flavonoids (anthocyanins, proanthocyanidins, flavanols, and flavonols), especially cyanidin-3-galactoside and cyanidin-3-arabinoside, as well as epicatechin units. High concentration of anthocyanins and flavones in *A. melanocarpa* is a prerequisite for these substances to have a positive therapeutic effect on immune system disorders [8,9]. The ascorbic acid contained in *A. melanocarpa* is also an antioxidant likely involved in many aspects of immune system activity [10].

*A. melanocarpa* is a fruit in which production is seasonal, so raw material processing is required to ensure its availability throughout the year [11]. In order to preserve the immunomodulatory activities of the extracts, it is necessary to harvest and store the fruit correctly. The well-known raw material preparation methods are drying and freezing. The work [12] demonstrates that the harvesting and storage method for raw materials affects their composition. Thus, during the dry raw material storage, proanthocyanidins—oligo- and polymerization products of monomeric flavan-3-ols under enzymes’ action—accumulate; in the frozen form, proanthocyanidins are 70–85% lower. Currently, there are no published data comparing the effects of *A. melanocarpa* extracts obtained from fruits harvested by different methods on the functional state and immune cell activity under simulated immunodeficiency conditions.

Thus, the aim of this study is to compare the antioxidant and protective properties in dried and frozen *A. melanocarpa* fruit extracts and to evaluate their effect on the functional state of peripheral blood immune cells in rats.

## 2. Results

### 2.1. Chemical Composition and Antioxidant Activity Analysis of Extracts

According to the raw material composition study, the highest anthocyanin content was found in freeze-dried aronia berries (58.6% more than the dried raw material). The frozen berries also had the highest ascorbic acid content, 3.8 times greater than dried raw material, as well as mono- and disaccharides (82.6% higher, respectively). Drying at room temperature allowed for greater preservation of flavonoids, 40% higher than indicators of frozen raw materials. The total phenolic number in the dried raw material was 24.7% higher (Table 1).

The flavonoid and phenolic component concentration did not change between extracts derived from dry and frozen source materials. A similar trend was seen for the other substances’ contents in terms of the original raw materials: the sugar content was 2.3 times greater in the extract from frozen raw materials, anthocyanins were 2.07 times higher, and ascorbic acid was 5.52 times higher, respectively (Table 1). The dry matter concentration of aronia propylene glycol extracts from frozen and dried raw material changed somewhat, being 13–16% and 11–13%, respectively. 

The activity of *A. melanocarpa* fruit extracts in DPPH (2,2-diphenyl-1-picrylhydrazyl) radical scavenging was also tested in this study. The findings correlated with total polyphenols, total anthocyanins, and ascorbic acid content. A stronger radical quenching agent usually resulted in a lower EC_50_ value.

*A. melanocarpa* extracts differed significantly in their DPPH radical scavenging ability. As shown in Table 1, DPPH uptake activity was higher in *A. melanocarpa* fruit extracts subjected to drying and decreased in fruit extracts subjected to freezing. The mean EC_50_ values for the *A. melanocarpa* fruit extract subjected to storage at low temperatures and to drying were 228.5 and 8.2 μg/mL, respectively. In comparison, the EC_50_ of Trolox is 29 μg/mL.

**Figure 1:**
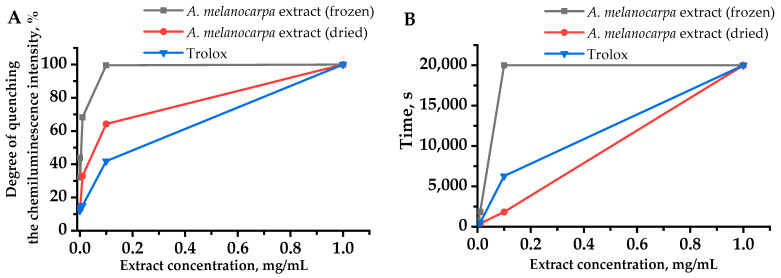
Dependence of TAR (total antioxidant reactivity) values (**A**) and TRAP (total reactive antioxidant potential) values (**B**) on the concentration of extracts obtained from the quenching of luminol-enhanced chemiluminescence.

In comparison with Trolox, the chemiluminescent activity analysis for the studied extracts revealed a pronounced ability to quench the chemiluminescence (CL) intensity. This ability was more pronounced for the extract from frozen raw materials, which was manifested by a higher CL intensity quenching (total antioxidant reactivity or TAR) (Figure 1A) and an increased latent period (total reactive antioxidant potential or TRAP) (Figure 1B), which is associated with the radical binding ability for the frozen aronia fruit extract.

### 2.2. Effect of Extracts on Rat Red Blood Cells Hemolysis in In Vitro Tests

**Figure 2:**
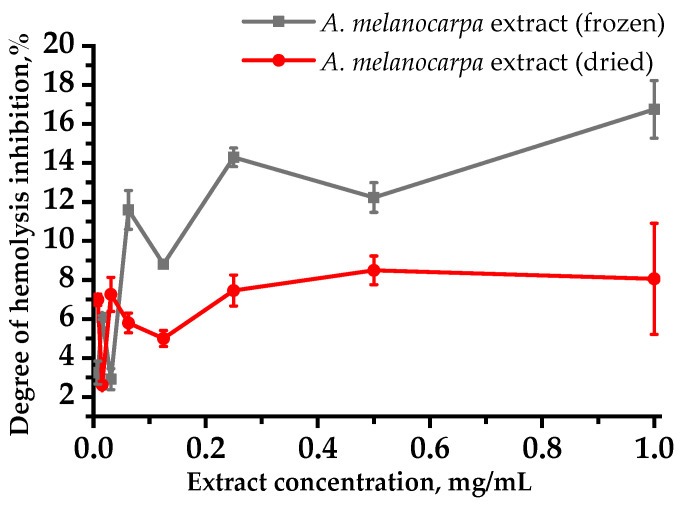
Dependence of red blood cell osmotic hemolysis on the aronia extract concentration on the model system. Values are expressed as percent inhibition (mean ± SD).

Figure 2 shows experimental results on the studied extracts’ ability to inhibit osmotic hemolysis, expressed in % relative to the control sample (without extracts) taken as 100%. These results indicate that the red blood cells (RBC) membrane resistance increases with increasing plant extract concentrations. When the extracts’ effects on RBC membranes were evaluated, it was discovered that the frozen aronia fruit extract was 5% more pronounced than dried fruit extract in stabilizing rat RBC membranes during osmotic hemolysis under in vitro conditions (Figure 2). There was a direct correlation between the hemolysis inhibition degree and extract concentration. The greatest membrane-stabilizing properties of the studied aronia fruit extracts were observed at the 1 mg/mL concentration, at which hemolysis processes were inhibited by 16% and 8%, respectively.

### 2.3. Determination of Cytoprotective Activity for Extracts from A. melanocarpa Fruits In Vitro

**Figure 3:**
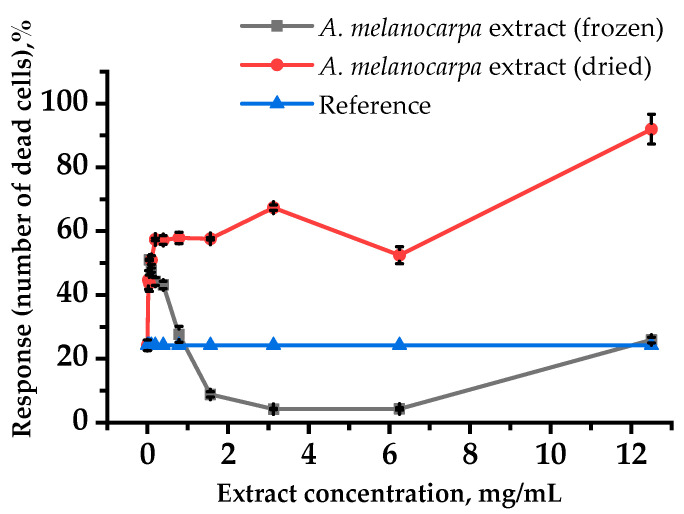
*A. melanocarpa* fruit extracts’ cytoprotective activity against the RPMI-1788 human lymphoblast cell line.

We investigated the propylene glycol extracts’ cytoprotective activity of aronia fruits with in vitro experiments on the RPMI-1788 cell line. Cyclophosphamide (CP) as a cytostatic drug inhibited culture viability. In control, under CP at a 1 mg/mL concentration, dead cells increased up to 43.7%. As seen in Figure 3, when the frozen aronia fruit extract was applied to the cell line incubation medium, the cell viability increased. Compared with the control, when dried aronia fruit extract was applied, the cell viability did not change, or even decrease. The data obtained demonstrates that frozen aronia fruit extract can protect cells from cytotoxic CP effects. Frozen aronia fruit extract showed cytoprotective properties in the whole concentration range studied, but the greatest effect, in which cell viability exceeded the reference values, was revealed in the range 0.195–6.25 mg/mL.

### 2.4. Hematological Analysis in Peripheral Rat Blood

Hematological studies are the most practical means to provide an integral assessment of the CP cytostatic immunosuppressive effect and the studied effect of aronia extracts on the blood system in in vivo studies. 

**Figure 4:**
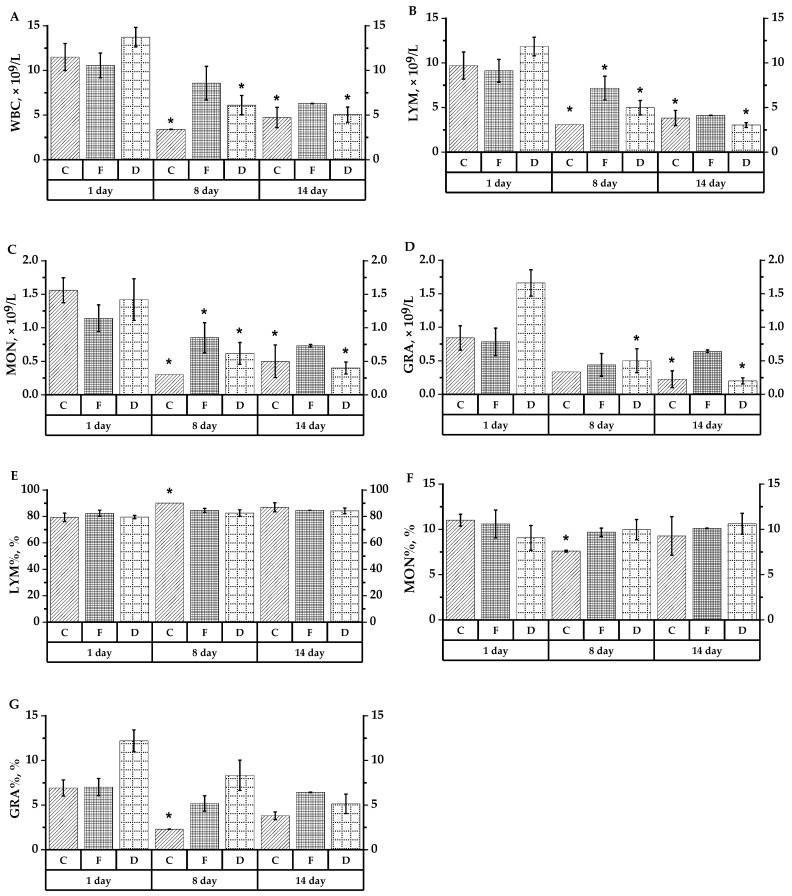
The main indices for hematological analysis of laboratory animals’ whole blood of groups C, D and F. (**A**) White blood count (WBC); (**B**) lymphocyte count (LYM); (**C**) monocyte count (MON); (**D**) neutrophil granulocyte count (GRA); (**E**) lymphocyte relative values from total leukocyte count (LYM%); (**F**) monocyte relative values from total leukocyte count (MON%); (**G**) granulocyte relative values from total leukocyte count (GRA%). * (*p* < 0.05)—reliability of differences in animal groups compared to 1 experimental day. The values are the means from three independent experiments ± SD.

Hematological studies revealed leukopenia after CP administration, most pronounced in the control group, where there was a 3.4-fold decrease in the white blood cell (WBC) number. In the experimental groups, the decrease in leukocyte count was less pronounced, 1.2- and 2.4-fold, respectively, after introducing the extracts from frozen and dried berries (Figure 4A). At the same time, a decrease in the absolute number of leukocyte subpopulations was observed: in the control group (group C), after CP injection, lymphocyte count (LYM) was 3.1 times lower, monocyte count (MON) 5.2 times lower, and granulocyte count (GRA) 2.5 times lower (Figure 4B–D). Lymphocyte count as LYM%, after CP injection, on the contrary, increased up to 90.1% instead of 79.3% (differences are significant at *p* < 0.05) (Figure 4E). Monocyte and granulocyte counts, i.e., immune cells capable of participating in phagocytosis, decreased after CP administration not only in absolute values, but also in relation to the total leucocyte count. Thus, in group C (or control group) there was a 1.45-fold decrease in MON% and a 3-fold decrease in GRA% (Figure 4F,G). In the experimental groups, the changes in leukocyte subpopulation ratio under CP action were less pronounced, but the decrease in absolute LYM, MON, and GRA values as compared to the initial level was statistically significant (*p* < 0.05). There was a 1.27- and 2.38-fold decrease in absolute LYM counts, a 1.34- and 2.29-fold decrease in MON, and a 1.77- and 3.32-fold decrease in GRA in the groups receiving *A. melanocarpa* propylene glycol extracts obtained from frozen (group F) and dried fruits (group D), respectively. The relative LYM% lymphocyte count in the experimental groups differed from baseline statistically insignificant, with a decreasing trend in group F and an increasing trend in group D. The percentage MON% in both experimental groups did not change when CP was administered, and GRA% decreased 1.36 and 1.46 times in the group using extracts from frozen and dried chokeberry fruits, respectively.

On day 7 following CP administration (day 14 of the experiment), WBC was reduced by 2.44, 1.68, and 2.95-fold in the control group and in the groups with administration of frozen and dried extracts, respectively (Figure 4A). LYM, MON, and GRA, similarly, in the same groups, were reduced 2.54, 2.21, and 3.89 times; 3.12, 1.56, and 3.55; and 3.65, 1.22, and 8.3 times, respectively (Figure 4B–D). LYM% and MON% percentages on day 14 were not statistically significantly different from the baseline, with a trend toward lower LYM% and higher MON% in the control group. GRA% on day 14 was reduced from the baseline by a factor of 1.8, 1.1, and 2.4 in groups C, F, and D, respectively (Figure 4E–G).

The obtained results demonstrate the positive effect of aronia extracts on the studied rat peripheral blood indices characterizing the immune system state. If we compare the two methods of preparing raw materials, the positive effect is more pronounced in the extract obtained from frozen raw material.

### 2.5. Analysis of Phagocytic Leukocyte Activity

**Figure 5:**
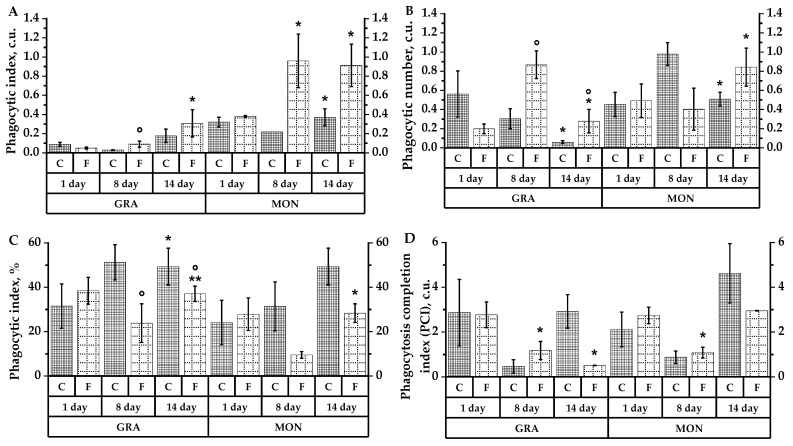
Indices of neutrophilic granulocyte and monocyte phagocytic activity in peripheral rat blood of groups C, D and F. Indices (phagocytic index (**A**); phagocytic number (**B**)) are expressed in units; phagocytic index (**C**) is expressed in %; phagocytosis completion index (**D**) is expressed in conventional units (c.u.). * (*p* < 0.05), ** (*p* < 0.01)—differences reliability compared to 1 experimental day; ° (*p* < 0.05)—differences reliability between animal groups compared to the control group.

The study revealed that the neutrophil granulocyte phagocytic index and peripheral blood rat monocytes in group C decreased in response to CP administration, that is, the uptake of *Escherichia coli* bacterial particles by immune cells decreased (Figure 5A). There was an increase in phagocytic index in neutrophil granulocytes and monocytes under the influence of frozen aronia fruit extract (*p* < 0.05). One week after immunosuppression modulation (day 14 of thae experiment), the phagocyte index value of granulocytes increased significantly in experimental group F (*p* < 0.05), to a lesser extent in group C. For monocytes, there was also an increase of bacterial particles per cell, by 16% in the control and 139% in the experimental group (*p* < 0.05).

Neutrophil granulocytes were more sensitive to cyclophosphamide’s suppressive action: in the control group, in response to cytostatic injection, the number of bacterial particles per active neutrophil (phagocytic number) reduced by 89% (Figure 5B). At the same time, in monocytes, we observed a tendency for this index to increase by 16% compared to the initial values. In the experimental group receiving frozen aronia fruit extract, against exposure to cyclophosphamide, the neutrophil and monocyte phagocytic number increased both in comparison with the initial values and with the control group. Compared with baseline values, granulocyte phagocytic count increased by 40% (with statistically significant differences from the control at *p* < 0.05), monocyte phagocytic count increased by 71%. On the 14th day, bacterial particle uptake activity increased significantly in groups C and F, with granulocyte activity significantly higher than in the control group (*p* < 0.05).

After CP administration, the percentage of actively phagocytized neutrophils and monocytes to total neutrophils (phagocytic index) in the experimental group did not differ from the initial values, whereas in the control group, the percentage of phagocytized neutrophils increased by 57% and monocytes increased by 56% (Figure 5C). A reduction in phagocytic neutrophil and monocyte counts was observed in group F on day 14, one week after CP delivery. It was comparable to both baseline levels and control group C (Figure 5), which was most likely attributable to the cytostatic’s delayed immunosuppressive effects.

In control group C, the phagocytosis completion index (PCI) of granulocytes decreased 6.1-fold and monocytes 2.4-fold in response to the cytostatic (Figure 5D). Modulation of immunosuppression against the administration of frozen aronia fruit extract also caused a decrease in granulocyte and monocyte PCI compared to the initial indices (*p* < 0.05). However, the neutrophil and monocyte PCI in group F was higher compared to group C. 

### 2.6. Spontaneous and Zymosan-Induced Chemiluminescent Neutrophil Activity Analysis

**Figure 6:**
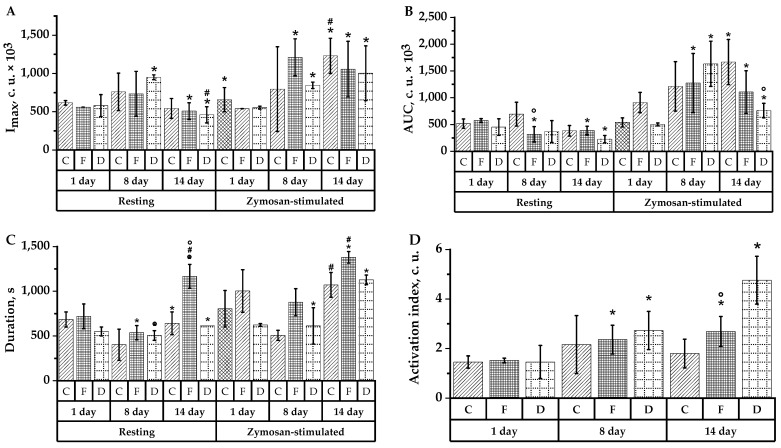
Spontaneous and zymosan-stimulated chemiluminescence indices of rat neutrophils of groups C, D and F: (**A**) CL intensity (I_max_); (**B**) area under CL curve (S); (**C**) time to reach maximum CL intensity (T_max_); (**D**) activation index (I_act_). * (*p* < 0.05)—reliability of differences in animal groups compared with 1 experimental day; # (*p* < 0.05)—differences reliability in animal groups compared with 8 experimental days; ° (*p* < 0.05)—differences reliability between animal groups compared with the control group.

The analysis of luminol-dependent CL found that in response to cyclophosphamide administration, the activation index increased relative to intact data significantly in all groups—3.27-fold in group D, 1.76-fold in group F, and 1.23-fold in group C (*p* < 0.05). Thus, on day 8, the activation index was 1.49-fold higher in group F and 2.64-fold higher in group D compared with the control (*p* < 0.05). On day 7 following CP administration, the index remained at the highest level in rats receiving dried aronia fruits—relative to intact values, 1.87-fold higher in group D and 1.48- and 1.54-fold higher in the control and group F, respectively (Figure 6D).

The latency to maximal chemiluminescence of a spontaneous CL response was significantly accelerated one day after CP treatment to animals in the experimental groups compared to intact values (*p* < 0.05) (Figure 6C). In contrast to the control group activity data, group F and group D exhibited a 1.80-fold (*p* < 0.05) and 1.22-fold decrease in the total active oxygen radicals (area under the chemiluminescent curve), respectively (Figure 6B), and a 1.31-fold (*p* < 0.05) increase in the maximum respiratory burst—in group F and group D—1.64 times (*p* < 0.05), which exceeded the control value’s day 8 CL intensity (Figure 6A).

Parameters zymosan-induced CL of neutrophils 24 h after immunosuppression modeling revealed a 2.24 and 1.53-fold increase of maximal respiratory burst in groups F and D, respectively (Figure 6A), and a 1.4 and 3.25-fold increase of total reactive oxygen radicals in groups F and D, significant compared with intact values (*p* < 0.05), moreover, the highest area under the curve was observed in group D (Figure 6B). The time to the zymosan-induced curve maximum significantly decreased in group F (*p* < 0.05) (Figure 6C).

The analysis of spontaneous luminol-dependent CL neutrophil parameters in experimental rats 7 days after they received cyclophosphamide revealed a prolonged time to the maximum CL intensity. In group F, it increased significantly relative to day 8 by 2.17 times, whereas in the control and group D—in 1.59 and 1.21 times, respectively (Figure 6C); a decrease in the maximum respiratory burst relative to the data of day 8 in the control and in group F by 1.4 and 1.44 times, respectively, and by 2.07 times in group D (*p* < 0.05) (Figure 6A). Additionally, there was an increase in the total number of active oxygen radicals in group F by 1.22 times, but significantly less in groups F and D compared with intact values (Figure 6B).

The CL parameter analysis on day 14 revealed that zymosan induction consistently increased neutrophils’ response time to stimuli in all groups, compared to intact values (Figure 6C). In experimental groups, increased relative to intact indicators blood neutrophil’s ability to enhance secondary reactive oxygen intermediate (ROI) production in response to in vitro stimulation in all groups studied, compared to day 8—in the control group (*p* < 0.05) (Figure 6A). The area under the zymosan-induced CL curve increased in all groups (*p* < 0.05), increasing to the greatest extent—3.1-fold—in the control group relative to intact values (*p* < 0.05) (Figure 6B).

Relative indices characterizing primary and secondary ROI production by blood neutrophils under stimulation with zymosan in vitro demonstrate that during luminol-dependent CL, the maximal CL intensity increased relative to the spontaneous one in the rats treated with the frozen aronia fruit extract by 64.9% and 107.7% on days 8 and 14 of the experiment. The total active oxygen radicals increased maximally on day 8 in groups F and D and on day 14 in group C.

Thus, the increase in the relative indexes of zymosan-stimulated CL was observed to the greatest extent in the group of rats treated with the frozen aronia fruit extract.

### 2.7. Cytokine Analysis

To identify specific cytokines involved in inflammatory and immune responses produced by immune cells, we used the MILLIPLEX MAP Cytokine/Chemokine Magnetic Bead Panel protocol to screen cytokines in leukoconcentrates obtained from rat blood in the study groups, and analyzed the data block. The data obtained allowed us to evaluate the functional status in regards to the immune rat blood cells under CP-induced immunosuppression according to the leukocyte ability to produce cytokines. The values for cytokines IL-10, IL-12P70, IL-15, IL-17a, IL-1α, IL-1β, IL-2, IL-3, IL-6, and TNFα were not determined, as they were below the threshold determination values for this research method.

On day 8 of the experiment, in groups F and D, there was a drop in colony-stimulating factors granulocyte colony-stimulating factor (G-CSF) and granulocyte-macrophage colony-stimulating factor (GM-CSF), as well as the cytokine IL-7, which induces hematopoiesis (Table 2). On the 14th day, the G-CSF level significantly increased 1.13 and 2.43-fold in groups C and F, respectively, and decreased 2.25-fold in group D. IL-7 level increased 1.81-fold in controls on day 14, and 1.27-fold in group D.

It was found that under the effect of cyclophosphamide, growth factors such as epidermal growth factor (EGF), transforming growth factor alpha (TGFα), and VEGF increased in the control group. Growth factors such as fibroblast growth factor-2 (FGF-2), VEGF, platelet-derived growth factor-AA (PDGF-AA), and PDGF-AB/BB increased in the control group and group D, and were significantly higher than in group F.

FMS-related tyrosine kinase 3 ligand (fLT-3L), which is a major growth factor for dendritic cells, increased in the control group after CP injection on day 8 and to an even greater extent on day 14. This index decreased in group F in response to CP administration, then increased on day 14. In group D, the fLT-3L level increased slightly after CP administration (day 8 of the experiment) compared with the initial level and recovered on day 14. It was significantly lower than in the control group.

The concentration of proinflammatory cytokine interferon gamma (IFN-γ), which activates the effector macrophage functions, increased in response to CP administration in the studied groups, more so in group D, which is usually observed in the initial immune deficiency disease period. Its production increased on day 14 in the control group and decreased in groups F and D. 

The chemokine IFN-γ-inducible protein (IP-10) level increased on day 8 in group C, decreased in group F below the detectable threshold, and remained unchanged in group D (Table 2). On day 14, it remained unchanged at the elevated level in groups C and D, and increased in group F.

IFNα2, which possesses pronounced antiviral, antiparasitic, and antiproliferative activity, increased in response to CP administration relative to intact values in groups C and D, but was significantly lower in group F.

On day 8 of the experiment, no change in eotaxin level was observed in the studied groups compared to the initial level, but it was significantly lower in group F than in group D (*p* < 0.01). On the 14th day, the eotaxin level increased in the control 1.42 times (*p* < 0.05), in group F—4.31 times (*p* < 0.001), and in group D it remained at the same level.

The neutrophil chemotactic factor IL-8 decreased on day 8 in groups C and D. In group F IL-8, it remained at intact values and increased on day 14 (*p* < 0.01).

In response to CP administration in groups C and D, there was an increase in macrophage-derived chemokine (MDC), which is usually expressed after mononuclear phagocyte differentiation from monocytes to macrophages [13], on day 8, with a slight increase in the control group and statistically significant in group D (*p* < 0.01). On day 14, the increase in MDC in group C was significantly different from the baseline level (*p* < 0.05), and MDC decreased slightly in group D. By contrast, in group F, MDC decreased on day 8 (significantly different from control) and increased on day 14.

Chemoattractant IL-8 tended to decrease in groups C and D under CP action, while remaining lower on day 14. In group F, this index significantly increased on day 14, by 10 times relative to the initial level.

The proinflammatory cytokines—macrophage inflammatory proteins (MIP1α and MIP1β) increased in control and group D on days 8 and 14 of the experiment (*p* < 0.05), but on day 14, MIP1α in group D was significantly (*p* < 0.05) lower than the control. In group F, these values were below the determination threshold on day 8 and increased on day 14.

The RANTES (regulated on activation, normally T-cell expressed, and secreted) chemokine (also known as CCL5) level increased on day 8 in group C, remaining at the same level on day 14. In group D, this index also increased on day 8 and became slightly lower on day 14. Group F RANTES on day 8 was below the determination threshold, but increased on day 14.

The anti-inflammatory cytokine IL-4 level increased in response to CP administration in groups C and D, and was significantly lower in group D than in the control group on days 8 and 14. IL-4 in group F was below the determination threshold on day 8, but increased on day 14 and did not differ from the control level.

Anti-inflammatory cytokine IL-13 levels in response to CP administration decreased in all groups, but increased slightly on day 14 in groups C and F.

The concentration of IL-9, a cytokine stimulating cell proliferation and preventing apoptosis, decreased 1.42 and 1.39-fold in experimental groups F and D, respectively, and increased 1.23-fold in group C. On day 14, the cytokine concentration increased 3.74-fold in group F, and decreased in groups C and D.

Fractalkine levels increased on day 8 in groups C and D, while in group F fractalkine decreased and was significantly lower, compared with group C. On the 14th day, this index increased in groups C and F, remaining unchanged in group D.

The monocyte-specific chemoattractant-1 (MCP-1) increased 2.18-fold on day 8 in group C, 1.34-fold in group D, and was below the determination threshold in group F. On day 14, MCP-1 further increased in groups C and F, decreased in group D, and was significantly lower than the control.

The interleukin-1 receptor antagonist (IL-1RA) as an endogenous anti-inflammatory agent decreased on day 8 in groups C and D, and was higher in group D than in group C. IL-1RA was below the determination threshold on day 1 and 8 of the experiment in group F. On day 14, this index fell in the control group while increasing in groups F and D and being higher than the control.

The soluble CD40 (sCD40) level significantly increased in the control group on day 8 and remained at the same level on day 14. In group D, this index did not change significantly during the experiment, within the reference values. In group F, sCD40 on day 8 after CP injection was below the determination threshold, and on day 14, it was significantly below the control.

## 3. Discussion

Chemical composition analyses of the initial raw material and propylene glycol extracts of the chokeberry have revealed that biologically active substances contained in the *A. melanocarpa* fruit are rather high. The content of such biologically active substances, such as ascorbic acid and anthocyanins, in general, determine the high antioxidant activity of the extract. The study has shown that compound classes, such as polyphenols, proteins, and polysaccharides, can affect the immune system by enhancing or suppressing immune reactions [2]. As described by Benvenuti et al. [14], reduced ascorbic acid in *A. melanocarpa* fruits is relatively low (13.1 mg/100 g raw weight), while the total polyphenols, on the other hand, are quite high and range from 192 ± 1.1 to 714.1 ± 7.4 mg/g dry weight [15,16,17,18,19,20]. The average total anthocyanin content in *A. melanocarpa* fruits is 460.5 mg/100 g raw weight. 

In this study, we examined the biologically active substance content of chokeberry fruits based on harvesting and storage methods, namely, dried and frozen raw materials. It was shown that drying decreased the biologically active compounds (mono- and disaccharides by 82.7%, anthocyanins by 58.6%, ascorbic acid by 281.9%) and antioxidant activity (in the extract concentration 0.1 mg/mL by TAR—by 35.3%, by TRAP—the latent period decreased by 18.17 s). However, drying the raw material increased the amount of retained flavonoids by 40% and phenolic compounds by 27%, as reflected in the antioxidant activity expressed in DPPH analysis. A similar effect was observed by Horszwald, Julien and Andlauer [21], who determined a higher total polyphenol content in *A. melanocarpa* after drying in a vacuum oven, especially at 60° C, then freeze-drying at low temperatures. The lower flavonoid content in frozen fruit extracts is seemingly due to the polyphenols’ degradation to some extent at low temperatures. It was also shown by Rauf et al. [12] that the harvesting and storage method of the raw material affects its composition. Thus, during the drying of the raw material, there is an increase of proanthocyanidins, while in the frozen state, proanthocyanidins are 70–85% lower. There is also data showing that the antioxidant activity depends on the harvesting method of the raw material. The authors’ work [21] confirms that, among other raw material processing methods, the lowest radical scavenging ability is noted in raw plant materials obtained after vacuum drying at high temperatures.

As shown by Benvenuti et al. [14], the radical scavenging DPPH activity values correlate significantly with the total polyphenol content. According to our data, the ability to trap DPPH radicals was higher in extracts obtained from dried raw materials, for which the total phenolic number of the original raw materials was higher than the original frozen raw materials. In extracts, the total phenolic number was the same. Simultaneously, the flavonoid content in the extracts from the dried raw material was 6.9% higher than in the extracts from the frozen raw material. However, in accordance with our results, the mean DPPH-radical uptake activity values did not correlate with the anthocyanins or ascorbic acid total (Table 1). According to Kalt et al. [22], the phenolic compounds and anthocyanin content correlated significantly with antioxidant activity, but ascorbate content, on the contrary, correlated negatively. Prior et al. [23] found that the ascorbic acid level was not a significant factor influencing the measured antioxidant activity of ORAC (oxygen radical absorbance capacity) by the APPH (2,2’-azobis(2-amidinopropane) dihydrochloride) method. It was shown [24] that L-ascorbic acid binds APPH radicals, leading to complete chemiluminescence quenching. However, it was revealed that the transformation by L-ascorbic acid formed in the observation dynamics leads to an increase in CL level, to the manifestation of the pro-oxidant properties. According to the data obtained in our work, the CL assay for frozen extracts revealed more pronounced quenching of free radicals APPH, up to 100%. In contrast to the DPPH assay method, chemiluminescent analysis makes it possible to evaluate the substance interaction reaction with APPH radicals immediately after staging the reaction and to study its dynamics. This method makes it possible to evaluate the contribution of unstable antioxidants such as ascorbic acid to antioxidant activity and to reveal their pro-oxidant properties. The enhancement of antioxidant properties of frozen chokeberry extracts compared to extracts from dried fruits revealed by the APPH method is due to the compounds present synergistically, in particular, to the higher ascorbic acid and anthocyanin content. The synergistic effect, or antioxidant compounds’ ability to reinforce each other, is consistent with previous findings [25].

The established membrane-protective activity may be due to the high anthocyanins, flavonoids, and other compounds contained in *A. melanocarpa* extracts [11]. The results presented in this study confirm the previously obtained data that the introduction of black chokeberry extract into the diet reduces lipid peroxidation in RBC [26]. A comparative analysis of membrane stabilizing effects under hypoosmotic conditions revealed a more pronounced protective effect of frozen fruit extracts (Figure 2), for which a higher content of the anthocyanins and ascorbic acid, and a higher antiradical antioxidant activity determined by the APPH method were found compared with dried fruits.

Research found that frozen aronia extracts on the RPMI-1788 lymphoblast cell line exposed to CP also showed cytoprotective properties. This suggests that higher antioxidant proanthocyanidins and ascorbic acid in the frozen *A. melanocarpa* fruit extract have a protective effect on cells against oxidative damage present in the CP cytotoxicity mechanism. Niedworok et al. [27] were the first to report that anthocyanins present in aronia extract protect rabbit RBC from CP-induced oxidative stress and protect healthy cells from reduced antioxidant enzyme activity during chemotherapy [28,29]. It was shown [30] that proanthocyanidins are highly active in radical scavenging ability, slow down low-density lipoprotein and lipid membrane oxidation induced by CP, and increase the antioxidant plasma activity. In addition, the protective effect of proanthocyanidins is associated with the chelation of redox-active metals as well as with some ROI captured. Several proanthocyanidins increase antioxidant enzyme activity and have a protective function against cell apoptosis. At the same time, it was shown in experiments that proanthocyanidins in 1–5 µg/mL concentrations have the best result, while higher concentrations have a toxic effect [31,32]. Ascorbic acid interacts with free radicals, binds them together, prevents oxidation and DNA damage, modulates antioxidant enzyme systems, and prevents macromolecule oxidation in cells, with inactive dehydroascorbic acid being formed. In addition, ascorbic acid easily reacts with CP to form a low-toxic salt [33]. Available data indicates that the polyphenolic compounds in chokeberries may also have significant cytoprotective potential, which has been described in a number of works preceding our study [34].

On the other hand, for dried aronia fruit extract, we did not find the cytoprotective effect range, because in the investigated concentrations of 0.1 mg/mL and higher, an extract did not decrease the number of dead cells compared to the control subjected only to CP, and in higher concentrations it increased the number of dead cells. Likely, this extract caused cell proliferation inhibition, which corresponds with the results described in [35]. We hypothesize that the dried aronia fruit extract components are responsible for the overall cytotoxicity as well as for the interactions between phenolic compounds and other co-extracted compounds. Previous research [36] found that polyphenolic compounds present in *A. melanocarpa* extract were active in vitro against a human leukemia cell line sensitive to HL60 promyelocytes and human promyelocytic leukemia cells resistant to doxorubicin (HL60/DOX).

According to the results of phagocytic activity analysis, in the control group after being modulated by immunosuppression, there was a 57% increase in the active granulocyte count and a 56% increase in the active monocyte count, while phagocytic sensitivity remained at baseline values. In response to CP delivery, the uptake activity of granulocytes and monocytes decreased. At the same time, 7-day prophylactic administration of frozen aronia fruit extract to rats and subsequent modeling immunosuppression increased the phagocytosed monocyte percentage by 71.4%. At the same time, the bacterial particle number in the latter was comparably increased (by 152.6%). Active granulocytes remained at the initial intact values, while the bacterial particle number in each neutrophil increased by 40%. This is evidence that the extract exhibited an activating effect on monocyte uptake activity and a protective effect on neutrophil granulocyte activity (Figure 5). Compared with the rats treated with cyclophosphamide, chemiluminescent granulocyte activity analysis revealed an increase in the blood neutrophils’ ability to enhance secondary ROI production in response to in vitro stimulation with zymosan, as evidenced by the cell activation index. The highest activation index was observed in the group receiving the dried fruit extract, then, in descending order, in the group with the frozen fruit extract and in the control. Within 7 days of administering the plant extract, ROI production levels normalized in all groups.

A cytokine status analysis of the rats treated orally with the frozen and dried aronia fruit extracts revealed anti-inflammatory effects more pronounced for the dried extract, manifested by an increase in the anti-inflammatory cytokines IL-4, IL-13, and IL-1RA, and a decrease in the proinflammatory cytokines MIP1α, MIP1β, and IFN-γ. The authors [37] obtained data that the oral administration of black chokeberry extract reduced proinflammatory cytokine levels of interleukin-6 (IL-6) and tumor necrosis factor (TNFα) in macrophages stimulated ex vivo with lipopolysaccharide, which is consistent with our results. The effects of these aronia extracts on pro- and anti-inflammatory cytokine levels are most likely due to certain polyphenols (cyanidin-3-arabinoside and quercetin), which are represented as a minor proportion of the common black chokeberry polyphenols [38]. It has been shown [39] that some of the plant extracts manifest their anti-inflammatory nature by inhibiting leukocyte transmigration from blood to tissue by reducing the expression of adhesive molecules on endothelial cells. According to Zapolska-Downar et al. [39], the expression of proinflammatory cytokine IFN-γ along with IL-2 demonstrates proinflammatory response of Th1 type T-cells. In other words, the data we obtained on the reduction of proinflammatory cytokines may also indicate a decrease in the activity of this type of immune cells.

## 4. Materials and Methods

### 4.1. Research Subject

Biologically mature fruits of the black chokeberry bush variety “Black pearl” *A. melanocarpa* were used in this study. Mature aronia fruits were collected and delivered during the harvesting season in September 2019 in the umbrella inflorescences with berries from the peasant farm “Shuiskiye yagody” (Russia). Average temperatures in the Ivanovo region during this time period were in the range of 5–26 °C during the day and 2–17 °C at night. The berries have a sour-sweet taste, are slightly astringent, are purplish-black in color, and covered with a grayish patina. The flesh is bright red, spherical in shape, with a maximum diameter of 0.9 cm and have an average weight of 1.2 g. The shrub was 4–5 years of age at the time of harvest. To study the harvesting method of the chokeberries and how storage influenced the extracts’ composition and properties, part of the raw material was dried in mild conditions at room temperature in a dark ventilated room. The second part was quickly frozen and stored in a freezer at −35 °C.

### 4.2. Obtaining the Extract

To obtain the extracts, the plant material was ground on a laboratory mill (LM 202, Russia) to a particle size ranging from 0.3 to 2 mm. An 85% solution of 1.3-propylene glycol was added, and the extraction process was carried out on a magnetic stirrer RCT basic (300 rpm) for 90 min at 50 °C. Given the moisture level of the raw material, a raw material to extraction solution ratio of 1:10 was employed when using dried chokeberry fruits. In the frozen fruits, the ratio was 1:7, respectively. The obtained extracts were centrifuged to separate coarse sediment at 4500 rpm and filtered over a Schott funnel with a 10–16 μm glass filter (class 4) at 20 mbar for additional purification and clarification.

The extracts and raw materials were determined by gravimetric drying in a thermostat at 105 °C [40] for moisture and dry matter content. The total sugar content in *A. melanocarpa* extracts was determined spectrophotometrically using a 5% aqueous phenol solution. A xylose-based reference solution was prepared [41].

The total phenol content in the *A. melanocarpa* extract was determined spectrophotometrically by adding Folin–Ciocalteu reagent and 7.5% sodium carbonate solution. The results were expressed in gallic acid, equivalent in mg per g, of dry extract (mg GAE/g) and recalculated per g of dry raw material [42].

The total flavonoid content was determined by the method [43] of using 2% AlCl_3_ ethanol solution. The flavonoid content was expressed in rutin equivalent mg per g of dry extract (mg Rut/g) and recalculated per g of dry raw material.

The anthocyanin content was determined spectrophotometrically [44]. The total anthocyanin content was expressed as mg equivalent of cyanidin-3-glucoside per g of dried or frozen fruit (mg CGlu/g).

The ascorbic acid content was determined by voltammetry using 3 M iron (III) chloride and 3 M 1.10-phenanthroline [45].

### 4.3. Determination of Antioxidant Activity and Biologically Active Substances in A. melanocarpa Extracts

The antioxidant activity of aronia extracts was studied by chemiluminescent and colorimetric methods that allow the determination of the substances’ ability to interact with free peroxide radicals APPH (Sigma Aldrich, USA) and DPPH (Alfa Aesar, USA), respectively. The chemiluminescent APPH method is described in [46] and adapted to the Lum-1200 luminometer (LLC Disoft, Russia) [24]. The results were processed on a personal computer using PowerGraph and OriginLab software. In the work of Lissi et al. [47], two approaches to measuring the total antioxidative capacity taking into account this feature of the curves are described—the TRAP and the TAR method. It is believed that TRAP reflects the number of antioxidants in the system, and TAR reflects its activity, i.e., the rate of the antioxidant interaction with radicals. The TRAP method is based on the measurement of the CL latency period. The TAR method was used to determine the value of CL intensity quenching.

The DPPH assay was done according to the method of Brand-Williams et al. [48] with some modifications. The stock solution was prepared by dissolving DPPH with methanol. Fruit extracts (150 mL) were allowed to react with 2850 mL of the DPPH solution for 30 min in a dark at room temperature. The absorbance for different concentration of extracts was then consecutively taken and measured at 517 nm. Results are defined as concentration of substrate that causes 50% loss of the DPPH activity and expressed in mg/mL fresh mass. 

### 4.4. Protective Activity of Extracts on the Osmotic Hemolysis Model for Rat Red Blood Cells In Vitro

The membrane-stabilizing activity in the extracts studied was assessed using a method initiating osmotic damage to the RBC membranes [49]. To obtain osmotic hemolysis, a hypotonic 0.3% sodium chloride solution was added to the RBC suspension. The extracts’ membrane-stabilizing effect was evaluated as the hemolysis inhibition percentage in relation to the parameters in the control (without adding the studied substance to the incubation medium).

### 4.5. In Vitro Experiments on the Human Leukocyte Cell Line RPMI-1788

Experiments were performed on a conditionally normal human leukocyte cell line—lymphoblasts RPMI-1788 obtained from the Russian collection of cell cultures of D.I. Ivanovskiy Institute of Virology (Moscow). The cells were cultivated on a RPMI-1640 medium with 10% fetal bovine serum (FBS), 1% essential amino acids, and antibiotic gentamicin was added [50].

#### 4.5.1. Determination of In Vitro Cytoprotective Properties

The RPMI-1788 cell suspension was prepared with a 10^5^-cell/mL concentration. Furthermore, 200 µL of the suspension was plated into a 96-well plate. To determine the cytoprotective effect [51], the toxicant CP at a 1 mg/mL concentration was added along with the test objects. The studied extracts and CP were not added to the growth medium during cell cultivation as a reference control. Only CP was added to the cell growth medium in the control group and incubated for 24 h. Each cell group was cultured in three replicates.

#### 4.5.2. Cell Staining and Counting

To determine the number of living and dead cells, a complete growth medium with fluorescent dyes was prepared at the rate of one well of 198 μL of the total growth medium, 2 μL Hoechst 33,342 (concentration of 1 mg/mL), and 0.5 μL of propidium iodide. Further, the culture liquid was replaced with a prepared growth medium with dyes and incubated for 45 min. After incubation, live and dead cells were counted on the guava easyCyte flow cytometer (Millipore, Germany).

### 4.6. In Vivo Experiments

The studies were performed on male Wistar laboratory rats with an induced decreased immune function (immunosuppression) due to CP administration. Against the induced immunodeficiency in rats, the effect of the studied aronia extractive substances on the animals’ immune status parameters, namely, the leukocyte count and their subpopulation ratio in peripheral blood, the immune cell functional state (chemiluminescent, phagocytic activity, cytokine production) was studied.

For 7 days, the rats prophylactically received water orally (control group, or group C) and *A. melanocarpa* propylene glycol extracts obtained from dried (group D) and frozen raw materials (group F) at a dose per dry substance of 50 mg/kg. CP was then administered once intraperitoneally at 25 mg/kg, and the extracts were continued to be administered orally for the next 7 days. Blood sampling was performed on day 1, before the experiment, and the results were taken as reference values, on day 8, i.e., one day following CP injection, and at the experiment completion, on day 14, i.e., 7 days following extracts administration against the induced immunosuppression.

#### 4.6.1. Hematological Tests

The number and subpopulation analysis of immune cells in peripheral rat blood was performed with an automatic hematology analyzer Mythic 18 Vet (Orphee SA, Switzerland) using a special reagent kit. In the rats’ white blood cells (WBC), lymphocyte count in absolute units and in relation to the WBC count to total leukocytes (LYM and LYM%, respectively), similarly, monocytes (MON and MON%) and granulocytes (GRA and GRA%) were determined.

#### 4.6.2. Functional Assessment of Neutrophil Granulocytes in the Rat Blood

Neutrophil chemiluminescence analysis detects cellular production of active oxygen radicals, including superoxide anion, singlet oxygen, hydroxyl radical, and hydrogen peroxide.

To assess the ROI formation by neutrophils, the cells isolated from experimental peripheral rat blood were registered by the method described in [52], without using a chemiluminescence activator (spontaneous or resting activity), and the neutrophil chemiluminescence signal response to activation by zymosan (activated activity).

The luminol-dependent neutrophil chemiluminescence kinetic curve was used to determine the following parameters: time to maximum CL (T_max_), intensity of maximum CL (I_max_), the area under the CL curve (S) for spontaneous CL, and CL induced by zymosan. The induced CL enhancement relative to spontaneous CL was assessed using area ratio under the chemiluminescence curve S_induced_/S_spontaneous_, thus determining the activation index (I_act_).

Phagocytosis, or uptake activity, was assessed by cytofluorimetry using a guava easyCyte flow cytometer, Millipore (Germany), only for the control group and the group receiving aronia extract from frozen berries (groups C and F). Inactivated *E. coli* bacteria labeled with fluorescein-5-isothiocyanate (FITC) (Sigma Aldrich, Germany) were used as an agent for phagocytosis according to the method described in [53,54,55]. The phagocytosis reaction was performed by incubating the leukoconcentrate (100 µL), prepared according to the method described in [52] and containing 1 × 10^6^/mL cells, with a 10 µL FITC-labeled suspension of *E. coli* bacteria (5 × 10^7^ bacteria in 1 mL) for 30 min and 120 min at 37 °C. The phagocytic number (PN) of neutrophils against FITC-labeled *E. coli* as the number of bacterial particles per neutrophil or monocyte that phagocytized, phagocytic activity (PA) as the number of active phagocytes that engulfed FITC-labeled bacteria to the total monocyte or neutrophil count, phagocytic index (PI) as the number of bacteria per neutrophil or monocyte on average, as well as the phagocytosis completion index (PCI) in conditional units, defined as the ratio of PN after 30 min to PN after 120 min [54,56].

#### 4.6.3. Cytokine Level Determination in Rat Leukoconcentrates

To determine cytokine levels, leukoconcentrates aligned with granulocyte counts (10^3^ granulocytes per μL) were subjected to RIPA Buffer lysis (Sigma Aldrich, USA). By multiplex analysis using the Milliplex Map Cytokine/Chemokine Magnetic Bead Panel reagent kit (Millipore, USA), changes in cytokine levels in leukoconcentrates were then determined on a MagPix instrument according to the reagent protocol.

### 4.7. Statistical Analysis

Statistical analysis was performed using nonparametric Mann–Whitney test in SPSS Statistics and Microsoft Excel 2016.

## 5. Conclusions

In the present study, *A. melanocarpa* fruit extracts prepared by two methods—freezing and drying in mild conditions at room temperature—were obtained. Their chemical composition, antioxidant, and protective properties were compared. It was revealed that the frozen *A. melanocarpa* fruit extract contains more anthocyanins, sugars, and ascorbic acid than the dry raw material extract and has a more pronounced antioxidant activity as measured by the capacity to bind free APPH-radicals. The dried raw material extract shows higher antioxidant activity by the ability to bind DPPH-radicals. Analyzing to a greater extent the antioxidant activity of extracts from dried and frozen chokeberry fruits depending on the biologically active substance content revealed that the extracts’ ability to trap DPPH-radicals is largely due to the polyphenols content and moderately due to the anthocyanins content. The ability to trap APPH-radicals is attributed to the total antioxidant properties of the extract, with all antioxidants in the extract working synergistically, including low-stable substances such as ascorbic acid.

The frozen aronia extract exhibited marked membrane-protective properties against rat RBC exposed to hypotonic solution and cytoprotective properties against RPMI-1788 human lymphoblast line cells exposed to cyclophosphamide at 1 mg/mL concentration. Based on the rat immunosuppression model induced by intraperitoneal injection of CP at 25 mg/kg, the extracts from dried and frozen chokeberry fruits at 50 mg/kg dose were found to accelerate the recovery of rats’ immune systems, normalize leukocyte count and their subpopulation ratio, improve monocyte and neutrophil phagocytic parameters, and enhance the functional immune cell reserve. Comparing the results of cytokine profile studies, we revealed the anti-inflammatory properties in chokeberry extracts that were more pronounced in dried extracts and showed an increase in anti-inflammatory cytokines IL-4, IL-13, and IL-1RA, and a decrease in pro-inflammatory cytokines MIP1α, MIP1β, and IFN-γ in leukoconcentrates. In addition, for several cytokines, there is a normalization of their quantity, which confirms that the immune cells’ functional state has improved and immunomodulatory properties were manifested.

## Figures and Tables

**Table 1:** Natural antioxidant content and DPPH radical scavenging activity in the original raw material and propylene glycol extracts of *A. melanocarpa* (per dry matter). ^1^—Total sugar content in xylose equivalent (Xy); ^2^—total phenolic number per gallic acid equivalent (GAE); ^3^—total flavonoids per rutin (Rut); ^4^—total anthocyanins per cyanidin-3-O-glucoside; ^5^—radical scavenging activity expressed as EC_50_ («efficient concentration»—value defined as the concentration of substrate that causes 50% loss of the DPPH activity).

Materials	Dry Matter		Total Sugars		Total Phenolic		Total Flavonoids		Total Anthocyanins		Ascorbic Acid		EC_50,_ mg/mL ^5^
	Raw Material %	Extract %	Raw Material mg Xy/g ^1^	Extract mg Xy/mL ^1^	Raw Material mg GAE/g ^2^	Extract mg GAE/mL ^2^	Raw Material mg Rut/g ^3^	Extract mg Rut/mL ^3^	Raw Material mg/g ^4^	Extract mg/mL ^4^	Raw Material mg/g	Extract mg/mL	
*Aronia melanocarpa* fruits, frozen	24.7	13–16	23.2	3.64	97	15.63	45	7.25	6.79	1.11	0.737	0.127	0.2285
*Aronia melanocarpa* fruits, dried	93.4	11–13	12.7	1.56	121	15.61	63	7.75	4.28	0.535	0.193	0.023	0.0082

**Table 2:** Cytokine level changes (pkg/mL) in leukoconcentrates of immunodeficient rats under the aronia extracts’ action. * (*p* < 0.05)—differences reliability in animal groups compared to day 1 of the experiment; # (*p* < 0.05)—differences reliability in animal groups compared to day 8; ° (*p* < 0.05)—differences reliability between animal groups compared to the control group; “ (*p* < 0.05)—differences reliability between animal groups compared to rats that received frozen aronia fruits.

Indicator	Group C			Group F			Group D		
	1st Day	8th Day	14th Day	1st Day	8th Day	14th Day	1st Day	8th Day	14th Day
EGF	1.85 ± 0.10	3.85 ± 0.59	4.48 ± 0.96	<3.2	<3.2	6.76 ± 1.45	<3.2	2.58 ± 0.73	1.85 ± 0.10
TGFα	0.05 ± 0.02	0.14 ± 0.02 *	0.19 ± 0.05 *	0.07 ± 0.02	<3.2	0.22 ± 0.11	0.01 ± 0.01	0.06 ± 0.02	0.03 ± 0.02
FGF-2	44.1 ± 6.01	55.07 ± 8.08	66.05 ± 5.98 *	15.27 ± 4.17 °	13.10 ± 2.00 °	41.40 ± 7.27 #°	42.67 ± 6.56 ”	51.27 ± 3.05 ”	51.11 ± 10.98
VEGF	69.98 ± 6.21	112.8 ± 24.21 *	99.67 ± 15.08	30.33 ± 7.32 °	36.94 ± 5.17 °	137.97 ± 17.22 *#	45.53 ± 3.55 °	92.11 ± 9.12 *”	58.59 ± 7.91 *°”
PDGF-AA	0.27 ± 0.12	0.47 ± 0.09	0.47 ± 0.07	0.11 ± 0.03	0.13 ± 0.03 °	0.47 ± 0.13	0.33 ± 0.12	0.25 ± 0.04 ”	0.36 ± 0.07
PDGF-AB/BB	52.87 ± 13.29	42.97 ± 4.14	59.19 ± 7.69	39.3 ± 7.66	21.2 ± 3.3 °	32.07 ± 4.87 #°	16.03 ± 2.48 °”	40.02 ± 4.54 *”	38.65 ± 5.7 *
Eotaxin	9.27 ± 0.79	10.88 ± 2.18	15.49 ± 1.4 *#	3.31 ± 1.10 °	2.64 ± 1.12	11.38 ± 1.68 *#°	7.2 ± 1.94	9.51 ± 0.98 ”	9.31 ± 0.81 °
G-CSF	19.46 ± 3.05	18.27 ± 2.05	20.61 ± 2.8 #	12.26 ± 0.03	6.77 ± 2.26	16.43 ± 6.42	19.83 ± 3.44	10.11 ± 2.12 *°	4.5 ± 2.24 *°
GM-CSF	0.97 ± 0.00	0.44 ± 0.23	0.59 ± 0.34	<3.2	<3.2	1.42 ±1.06	<3.2	0.13 ± 0.03	<3.2
IL-7	3.79 ± 1.3	3.44 ± 0.69	6.22 ± 1.16 #	4.98 ± 1.78	<3.2	5.91 ± 1.92	9.82 ± 4.5	2.44 ± 0.63	3.1 ± 1.04
IL-5	<3.2	0.11 ± 0.03	0.13 ± 0.04	0.04 ± 0	<3.2	0.35 ± 0.21	<3.2	0.07 ± 0.02	0.06 ± 0.02
fLT-3L	7.16 ± 0.86	9.90 ± 1.59	12.22 ± 1.70 *	5.26 ± 0.10	2.95 ± 0 °	9.08 ± 2.09 *	6.41 ± 1.15	7.65 ± 1.11 ”	6.35 ± 0.77 °
Fractalkine	80.93 ± 7.44	105.75 ± 15.3	131.39 ± 18.96 *	44.51 ± 6.42 °	35.75 ± 2.36 °	102.14 ± 19.74 *#	66.34 ± 8.59	77.44 ± 12.01	72.34 ± 8.65
IFNα2	37.78 ± 7.01	64.18 ± 10.48 *	47.48 ± 2.56	36.34 ± 3.12	16.39 ± 2.17 *°	33.71 ± 7.45 #°	17.63 ± 2.61 °”	47.32 ± 5.31 *”	28.34 ± 2.27 *#°
IFNγ	0.72 ± 0.23	1.47 ± 0.29	2.18 ± 0.41 *	1.08 ± 0.14 °	2.1 ± 0.1	1.96 ± 1.07	0.36 ± 0.02	1.00 ± 0.20	0.68 ± 0.32 °
IP-10	7.37 ± 1.05	15.46 ± 1.87 *	15.86 ± 2.17 *	6.32 ± 0	<3.2	16.82 ± 3.93	10.51 ± 0	11.31 ± 1.38	10.16 ± 2.12
TNFβ	<3.2	0.27 ± 0.09	0.3 ± 0.13	<3.2	<3.2	0.76 ± 0.45	<3.2	0.11 ± 0.02	<3.2
GRO	27.72 ± 2.34	33.99 ± 3.55	37.92 ± 2.36	15.11 ± 0.00	<3.2	41.14 ± 4.88	48.06 ± 6.18	27.71 ± 3.02 *	23.06 ± 3.09 *°”
IL-10	<3.2	0.53 ± 0.19	0.87 ± 0.58	<3.2	<3.2	2.06 ± 1.34	4.67 ± 1.27	<3.2	<3.2
MCP-3	13.74 ± 2.01	16.74 ± 2.7	21.34 ± 2.42	5.00 ± 2.17	2.83 ± 0.00	15.11 ± 3.32	10.59 ± 3.97	13.85 ± 2.39	12.63 ± 1.17 °
MDC	49.53 ± 3.76	59.4 ± 6.63	72.36 ± 7.08 *#	22.4 ± 8.46 °	12.83 ± 4.6 °	44.72 ± 8.05 #	34.75 ± 10.37	45.28 ± 7.28 ”	42.96 ± 4.86
IL-12p40	1.60 ± 0.10	4.42 ± 0.89	5.47 ± 1.44	<3.2	<3.2	8.63 ± 3.82	<3.2	2.40 ± 0.80	<3.2
IL-12P70	<3.2	1.62 ± 0.26	1.57 ± 0.79 #	<3.2	<3.2	2.98 ± 1.62	7.4 ± 0.92	<3.2	<3.2
sCD40	21.29 ± 2.58	40.71 ± 6.37 *	42.46 ± 7.54 *	30.62 ± 0.02	<3.2	38.17 ± 10.09	<3.2	30.38 ± 2.65	20.54 ± 2.96
IL-1RA	6.25 ± 2.11	5.74 ± 0.64	3.88 ± 1.16	<3.2	<3.2	36.57 ± 17	15.87 ± 9.11	11.6 ± 9.69 °	30.5 ± 11.01
IL-9	3.03 ± 1.06	3.74 ± 1.11	1.29 ± 0.35	0.75 ± 0.19	0.53 ± 0.09 °	1.98 ± 0.44	3.44 ± 0.79	2.48 ± 0.62	1.80 ± 0.29
IL-4	6.68 ± 2.81	24.1 ± 3.98	27.76 ± 4.57	4.1 ± 3.62	<3.2	25.83 ± 10.2	5.31 ± 2.42	10.68 ± 3.38 °	7.14 ± 2.76 °”
IL-13	7.0 ± 0.01	1.28 ± 0.23	2.23 ± 0.43	1.82 ± 0.39	<3.2	5.04 ± 0.01	<3.2	2.13 ± 0.47	1.67 ± 0.01
IL-8	1.94 ± 0.72	0.73 ± 0.14	1.4 ± 0.56	0.74 ± 0.18	0.93 ± 0.41	7.48 ± 1.28 *#°	6.18 ± 2.71	1.07 ± 0.37	1.05 ± 0.34 ”
MCP-1	1.37 ± 0.28	2.99 ± 0.49	4.35 ± 0.43 *	2.66 ± 0.18 °	<3.2	3.75 ± 1.13	1.53 ± 0.32	2.05 ± 0.32	1.41 ± 0.34 °
MIP1α	1.86 ± 0.57	5.39 ± 0.52 *	5.67 ± 0.75 *	1.28 ± 0.02	<3.2	5.03 ± 1.72	<3.2	3.12 ± 0.86	2.05 ± 0.77 °
MIP1β	1.66 ± 0.88	3.1 ± 0.98	3.73 ± 0.96	1.37 ± 0.59	<3.2	3.95 ± 1.54	<3.2	2.06 ± 0.56	0.78 ± 0.02
RANTES	4.17 ± 0.65	8.15 ± 1.0 *	8.45 ± 1.63	3.82 ± 0.67	<3.2	5.63 ± 2.06	3.3 ± 0.39	6.13 ± 0.48 *	4.12 ± 0.75 *

## Data Availability

The data will be provided by the authors upon request.
